# Development of a TaqMan RT-qPCR for the detection and genotyping of bovine viral diarrhea virus types 1, 2, and HoBi-like pestivirus

**DOI:** 10.1186/s12917-026-05685-4

**Published:** 2026-07-17

**Authors:** Yangyang Xiao, Qianqian Dong, Yifei Wu, Yang Yang, Jinchao Wang, Tao He, Qinghua Luo, Bowen Li, Tianying Chi, Ang Li, Jinliang Sheng, Faxing Wu

**Affiliations:** 1 College of Animal Science and Technology, Shihezi University, Shihezi, Xinjiang 832000 China; 2 Sub-center of the China Animal Health and Epidemiology Center for the National Center for Veterinary Culture Collection, Qingdao, Shandong 266032 China; 3 School of Biological & Chemical Engineering, Chongqing University of Education, Chongqing, 400067 China; 4 College of Life Sciences, Northwest A&F University, Yangling, Shaanxi 712100 China; 5 Animal Health and Epidemiology Center, National Animal Serum Bank, Qingdao, Shandong 266032 China; 6 Qingdao Key Laboratory of Modern Bioengineering and Animal Disease Research, Qingdao, Shandong 266032 China

**Keywords:** Bovine viral diarrhea virus, TaqMan probes, Detection, Differentiation, Multiplex RT-qPCR

## Abstract

**Background:**

HoBi-like pestivirus (HoBiPeV), as a newly discovered virus species in recent years, poses a serious threat to the global cattle industry due to its wide transmission and high pathogenicity. The genetic sequence of HoBiPeV exhibits significant differences when compared to the known strains of BVDV-1 (*Pestivirus bovis*) and BVDV-2 (*Pestivirus tauri*).Current detection methods are typically limited to identifying BVDV-1 and BVDV-2, and are unable to effectively differentiate HoBiPeV from the other two genotypes. To facilitate the rapid detection and differentiation of various genotypes, this study developed a pair of universal primers along with three TaqMan probes based on the 5’ UTR sequences of the BVDV-1, BVDV-2, and HoBiPeV available in GenBank. A one-step multiplex TaqMan RT-qPCR detection method was developed.

**Results:**

The results showed that this method could specifically detect the three genotypes of BVDV without cross-reactivity. It exhibited high sensitivity, with a minimum detection limit of 1 × 10¹copies/µL. The amplification efficiencies were 104.6%, 102.9%, and 117.7%, respectively, demonstrating a strong linear relationship (R^2^ > 0.99). This method also showed excellent intra-assay and inter-assay reproducibility, which facilitates accurate identification and detection of the three genotypes. In clinical sample testing, the present assay showed higher detection rates for all three genotypes than previously reported methods, particularly showing a notable sensitivity advantage in the detection of BVDV-1.

**Conclusions:**

This method not only significantly enhances the detection rate of HoBiPeV but also reveals the potential prevalence of this genotype in cattle populations, providing valuable insights for updating existing epidemiological databases and informing the selection of vaccine strains. The method is applicable in areas such as the detection of raw materials and intermediates during vaccine production, quality control of dairy products, and large-scale virus monitoring. It also exhibits broad application value in epidemiological investigations and the prevention and control of viral diseases in cattle farms.

## Background

Bovine viral diarrhea virus (BVDV)is a single-stranded RNA virus with a genome length of approximately 12.3 kb, exhibiting significant antigenic and genomic diversity [1]. Based on phylogenetic analysis of the 5’ UTR, N^pro^, NS3,NS5B and E2 regions of the genome, BVDV is classified into three main genotypes: BVDV-1 (*Pestivirus bovis*), BVDV-2 (*Pestivirus tauri*), and Hobi-like pestiviruses (HoBiPeV, *Pestivirus brazilense*) [2–5]. Currently, 24 sub-genotypes of BVDV-1 (BVDV-1a to -1x) and 5 sub-genotypes of BVDV-2 (BVDV-2a to -2e) have been described [6].BVDV-1 was first identified in North America in 1946; however, retrospective studies have demonstrated that the virus had already been circulating in multiple regions worldwide prior to its initial identification [7].BVDV-2 was genetically differentiated from BVDV-1 through studies conducted in the United States in 1989. Subsequent retrospective analyses of historical samples from different continents revealed that BVDV-2 had been circulating outside the United States before its formal classification [8]. HoBiPeV, a recently identified genotype in South America, Europe, and Asia, exhibits significant genetic differences from the known BVDV-1 and BVDV-2 genotypes [9].

BVDV has a broad host range, and multiple animal species, including cattle, pigs, sheep, deer, camels, and other wildlife, are susceptible to infection [10].Moreover, significant differences in pathogenicity and clinical manifestations have been reported among different BVDV genotypes [11]. Additionally, BVDV can be vertically transmitted through the placenta. Cows infected with BVDV can give birth to persistently infected (PI) animals that are tolerant to the virus [12].A subset of PI animals can reach adulthood similarly to healthy animals, but their performance in relation to growth, development, and reproduction are significantly compromised when compared to healthy counterparts. Moreover, these animals continuously shed the virus into the environment throughout their lifetime, making them a critical source of infection within cattle herds [13].

Currently, the diagnostic methods for BVDV mainly include molecular biological assays, antigen detection, and serological tests. Molecular techniques such as reverse transcription-polymerase chain reaction (RT-PCR) and loop-mediated isothermal amplification (LAMP) can directly detect viral nucleic acids. Antigen detection methods, including immunofluorescence assays and antigen enzyme-linked immunosorbent assays (ELISA), are used to detect viral proteins. Serological methods, such as virus neutralization tests and antibody ELISA, are primarily used to detect host antibodies [14–16]. Although virus isolation can be used for viral identification, it usually requires cell culture conditions, is time-consuming, and demands high laboratory standards, making it unsuitable for routine clinical diagnosis.In addition, serological methods may yield false-negative results in PI animals due to immune tolerance [17–19]. Therefore, there is an urgent clinical need to develop new diagnostic technologies that are faster, more sensitive, and capable of viral genotyping. Given the significant genetic diversity of BVDV, molecular biological methods have emerged as the primary approach for BVDV typing and have become essential tools for the early diagnosis of BVDV infection. Various PCR methods, such as nest PCR, multiplex diagnostic PCR, and quantitative PCR(qPCR), exhibit high stability and efficiency [20, 21].Particularly, qPCR is widely used in the accurate typing and epidemiological study of BVDV due to its advantages in automation level, operational simplicity, high sensitivity and specificity, and user-friendly data analysis. This method effectively prevents cross-reactivity with closely related viral strains, such as the classical swine fever virus (CSFV) and the border disease virus(BDV). It is applicable for detecting early-stage infections in cattle and screening persistently infected animals [22]. BVDV is an RNA virus whose genome may undergo nucleotide substitutions as well as homologous or non-homologous recombination during replication [23].These genetic features may contribute to the complexity of its prevention and control.It has been reported that BVDV may introduce point mutations during each replication cycle.Analysis of 5’UTR sequences of BVDV revealed a mean evolutionary rate of 9.3 × 10^− 3^ substitutions/site/year for the investigated sequences. Moreover, evolutionary rates of 5.9 × 10^− 4^ and 1.26 × 10^− 3^ substitutions/site/year have been reported for the 5’UTR and E1-E2 regions, respectively [23–25].Point mutations accumulating (genetic drift) and recombination events have driven the continuous diversification of genetic subtypes, resulting in high intra-genotypic variation.Currently, there is no internationally standardized genetic subtyping system for BVDV. Differences in the target gene regions and subtyping methods used across studies have resulted in inconsistent classification, and in some cases the same strain has been assigned conflicting subtypes.Most existing diagnostic methods focus on classification at the genotype (BVDV-1 and BVDV-2); subtype identification generally relies on sequencing followed by phylogenetic analysis. In addition, currently available assays show limited capacity to simultaneously detect and differentiate BVDV-1, BVDV-2, and HoBiPeV, particularly in the context of mixed infections and high genetic variability.The lack of rapid, sensitive, and high-throughput tools for genotype-level detection undermines effective surveillance, compromises data comparability, and impedes the timely implementation of control measures [7].

Therefore, this study utilized Primer Premier 5 to design a pair of universal primers targeting the conserved 5’UTR shared by all three genotypes, along with three specific TaqMan probes for BVDV-1, BVDV-2, and HoBiPeV respectively. A multiplex TaqMan RT-qPCR detection method was established, demonstrating rapid, sensitive, and specific performance, thereby enabling the simultaneous detection and differentiation of BVDV-1, BVDV-2, and HoBiPeV. This method holds significant practical value for large-scale viral surveillance, epidemiological investigations, vaccine production, and dairy quality control, particularly for the prevention and control of viral diseases on cattle farms.In addition, this method provides the technical basis for developing unified and practical subtype identification standards, which helps reduce typing discrepancies, improve consistency in epidemiological comparisons, and optimize BVDV monitoring and control strategies.

## Results

### Primer and probe designing

**Fig. 1:**
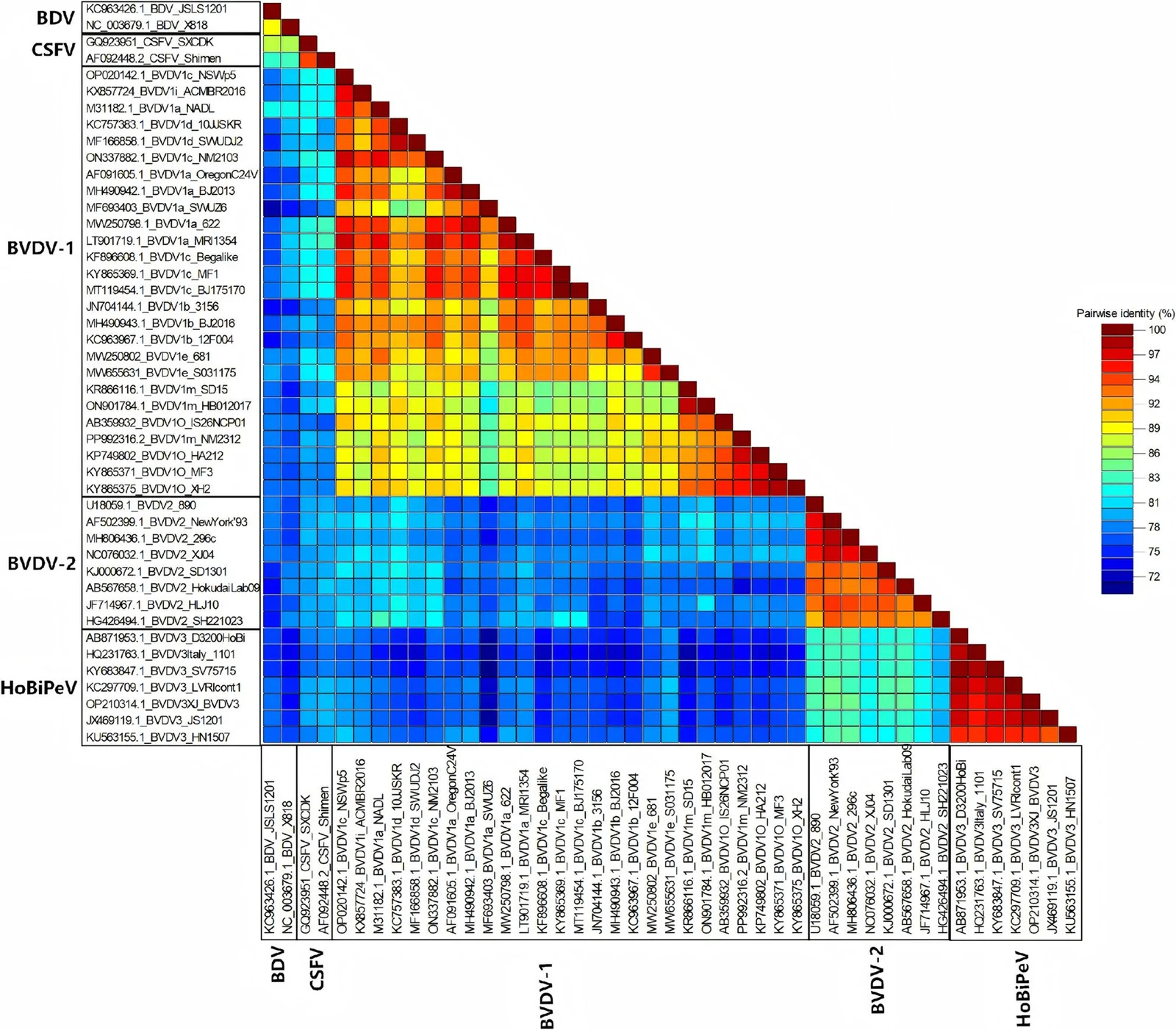
Comparative 5' UTR sequence homology of BVDV-1, BVDV-2, and HoBiPeV.Pairwise nucleotide sequence identity values were calculated based on 5' UTR sequences and are presented as a triangular heatmap. Each colored square represents the degree of sequence homology between two viral strains. The color scale on the right indicates increasing nucleotide identity from low (blue) to high (red), illustrating the genetic relatedness and divergence among the three pestivirus species

**Fig. 2:**
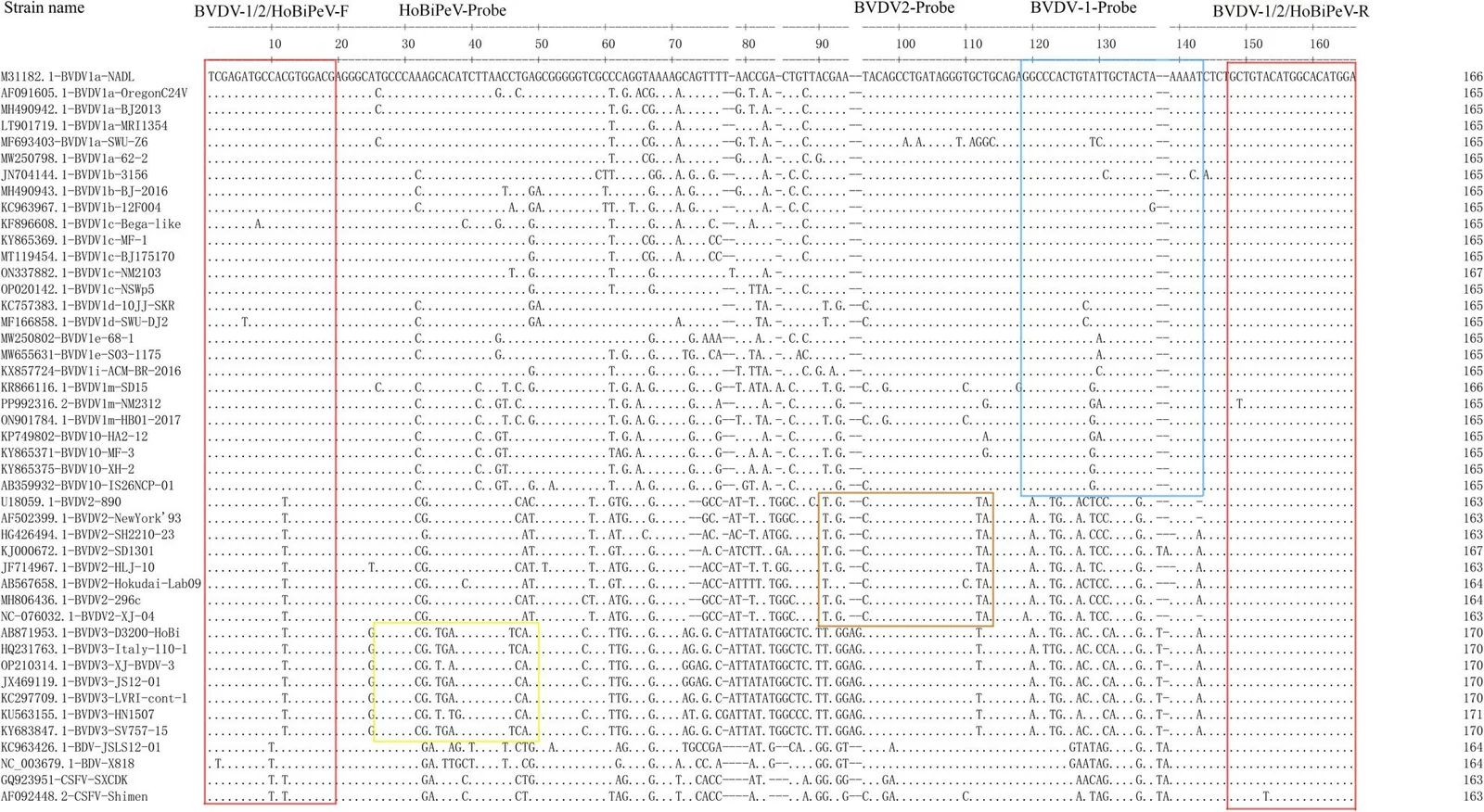
Nucleotide alignment of BVDV-1、BVDV-2、HoBiPeV. Conserved and variable nucleotide positions among the three viruses are shown. The binding regions of primers and TaqMan probes used in the multiplex RT-qPCR assay are highlighted with boxed areas, indicating the target sites selected for assay design

The 5’UTR nucleotide sequences of 26 BVDV-1 reference sequences, 8 BVDV-2 reference sequences, and 7 HoBiPeV reference sequences were retrieved from the NCBI database. The selected reference sequences span early to recent isolates to capture temporal evolutionary dynamics(1963–2023). Priority was given to strains circulating in China and neighboring regions, and representative strains of all major known sub-genotypes were included where possible. In particular, multiple epidemiologically relevant BVDV sub-genotypes were selected (BVDV-1a, 1b, 1c, 1d, 1e, 1i, 1 m, 1o; BVDV-2a, 2b, and 2c) to maximize sequence coverage.Sequence alignments were performed using MegAlign, with emphasis on recently circulating HoBiPeV reference strains, to ensure that primer and probe designs encompass genotypic sequence diversity and to improve the assay’s ability to detect newly emerging variants. Homology comparison of the reference sequences showed that the selected 5’UTR amplification regions of BVDV-1, BVDV-2, and HoBiPeV have homologies ranging from 72% to 100% (Fig. 1.). Based on this genomic framework, the 5’UTRs of BVDV-1, BVDV-2, and HoBiPeV were aligned with the 5’UTR sequences of CSFV and BDV to assess potential cross-reactivity of the designed primers and probes with non-target viruses (Fig. 2.). As shown in Fig. 2, BVDV-1/2/HoBiPeV F and BVDV-1/2/HoBiPeV R exhibited high sequence conservation across reference strains of BVDV-1, BVDV-2, HoBiPeV, as well as CSFV and BDV. BVDV-1-Probe, BVDV-2-Probe, and HoBiPeV-Probe also exhibited high sequence conservation within their respective genotypes but clearly mismatched to the other two genotypes (primer sequences are listed in Table 1). Collectively, these results indicate that the primer-probe combinations enable reliable differentiation of BVDV-1, BVDV-2, and HoBiPeV at the genotype, while avoiding cross-reactivity with CSFV and BDV.

**Table 1:** Primers and TaqMan probes used in the multiplex RT-qPCR assay

Primers and Probes	Reference	Sequence(5′→ 3′)	Position	Length (bp)
BVDV-1/2/HoBiPeV-F	This study	TCGAGATGCCAYGTGGACGA	225-244^a^ 227-245^b^ 176-195^c^	166 bp 164 bp 171 bp
BVDV-1/2/3-R		CTCCATGTGCCATGTACAGC	372-391^a^ 372-390^b^ 327-346^c^	
BVDV-1-P		HEX-ATTTTTAGTAGCAATACAGTGGGCC-BHQ1	343-367^a^	
BVDV2-P		ROX-CTACACCCTATCAGGCTGTGTCCA-BHQ2	316-339^b^	
HoBiPeV-P		FAM-TGCCCACGGTGAATCTTAACTCAAG-BHQ1	137-161^c^	
BVDV-1/2-TF		GAAGGCCGAAAAGAGGCTA		
HoBiPeV-TF		ACTAGTGGTAGCAGTGAGCTCCT		
BVDV-1/2/HoBiPeV-TR		CTCCATGTGCCATGTACAGC		
Pesti-qF	Mari et al. (2016)	GATGCCATGTGGACGAGGGC	229-248^a^ 233-252^b^ 116-135^d^	160 bp 156 bp 164 bp
Pesti-qR		CATGTGCCATGTACAGCAGAG	368-388^a^ 368-388^b^ 259-279^d^	
BVD1-Pb		FAM-CAATACAGTGGGCCTCTGCAGCA-TAMRA	341-363^a^	
BVD2-Pb		VIC-GTGGCGTTATGGACACAGCCTG-BHQ2	307-328^b^	
HoBiPeV-Pb		TexasRed-ATCAGGCTGTACTCCCAAAG-BHQ2	200-219^d^	

^a^Oligonucleotide position is referred to the sequence of BVDV-1 strain NADL (GenBank accession no. M31182)

^b^Oligonucleotide position is referred to the sequence of BVDV-2 strain New York (GenBank accession no. AF502399)

^c^Oligonucleotide position is referred to the sequence of HoBi-like pestivirus strain Th/04_KhonKaen (GenBank accession no. NC_012812.1)

^d^Oligonucleotide position is referred to the sequence of HoBi-like pestivirus strain Italy-1/10 − 1 (GenBank accession no. HQ231763) 

### Establishment of standard curves

**Fig. 3:**
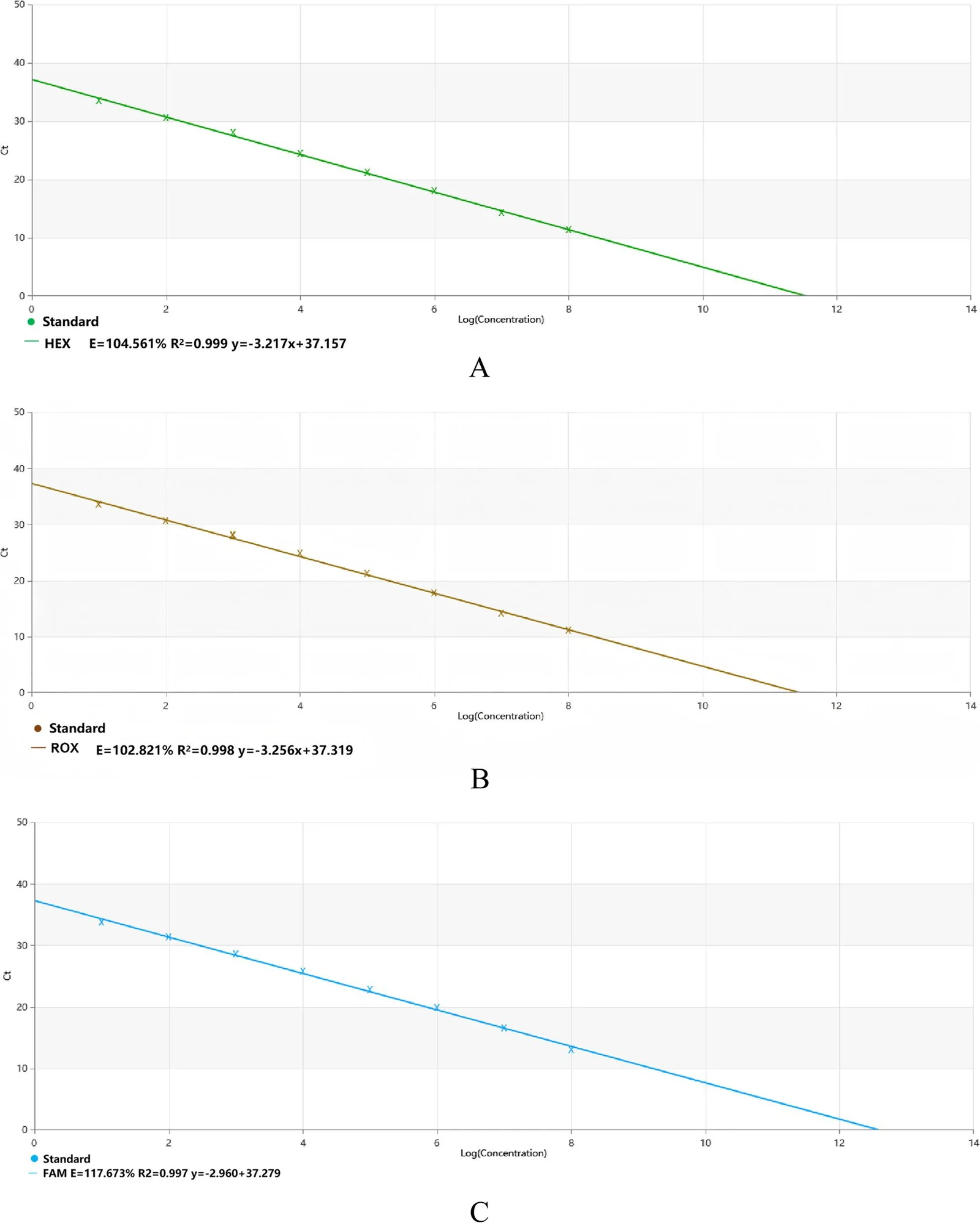
Multiplex RT-qPCR standard curve. **A **BVDV-1 5'UTR positive control; **B** BVDV-2 5'UTR positive control; **C** HoBiPeV Standard curves were generated using ten‑fold serial dilutions of plasmid standards containing the 5′ UTR fragments of **A** BVDV‑1, **B** BVDV‑2, and **C** HoBiPeV. The concentration range of the plasmid standards was 1 × 10^8^ to 1 × 10^1^ copies per reaction. A strong linear correlation was observed between the logarithm of template copy number and Ct values, demonstrating the quantitative performance of the assay

 A 10-fold serial dilution of mixed in vitro-transcribed RNA standards derived from representative reference sequences of BVDV-1, BVDV-2, and HoBiPeV 5’UTR regions was prepared with concentrations spanning from 1 × 10^1^ to 1 × 10^8^ copies/µL and used as templates for amplification.These RNA standards were generated from plasmids containing the target regions and were used as quantification standards for assay development and evaluation of amplification efficiency.Multiplex TaqMan RT-qPCR amplification was performed according to the optimized reaction system and protocol.A standard curve was established with the Ct value as the vertical axis and the logarithm of the RNA copy numbers as the horizontal axis. The results indicated that BVDV-1 exhibited a standard curve equation of y=-3.217x + 37.157 with a correlation coefficient (R^2^) = 0.999 and amplification efficiency (E) = 104.6% (Fig. 3.A.). For BVDV-2, the standard curve equation was y=-3.256x + 37.319, with R^2^= 0.998 and E = 102.9% (Fig. 3.B.). The standard curve equation for HoBiPeV was y=-2.96x + 37.279 with R^2^ = 0.997 and E = 117.7% (Fig. 3.C.).Overall, these results demonstrate that the developed multiplex TaqMan RT-qPCR assay provides robust, efficient, linear amplification across all in vitro-transcribed RNA standards representing each BVDV species, thereby supporting its suitability for further analytical and diagnostic evaluation.

### Specificity of the multiplex TaqMan RT-qPCR

**Fig. 4:**
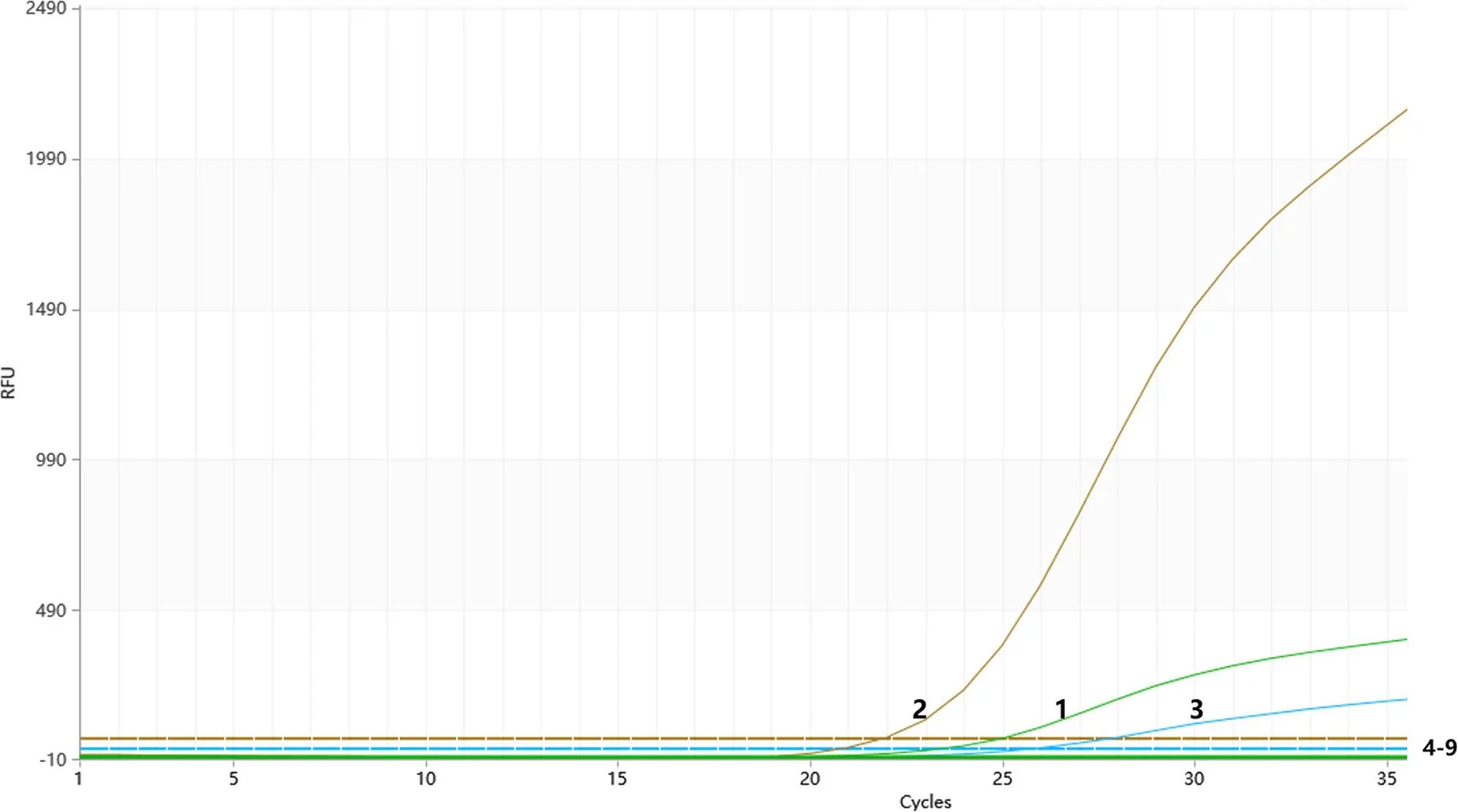
Assay specificity of the Multiplex RT-qPCR. 1: BVDV-1; 2: BVDV-2; 3: HoBiPeV ; 4: BCoV; 5: BRV; 6: BPIV3 7:BDV 8:CSFV ; and 9: N.The N denotes negative controls used in this study. Amplification curves were obtained using nucleic acid templates from BVDV-1, BVDV-2, HoBiPeV, BCoV,BRV, BPIV3, BDV, CSFV, and N. Specific fluorescence amplification signals were observed only for BVDV-1, BVDV-2, and HoBiPeV, whereas no amplification curves were detected for nontarget viruses or the negative control, demonstrating the high specificity of the multiplex RT-qPCR assay

To evaluate the specificity of the developed multiplex TaqMan RT-qPCR assay, RT-qPCR was performed using as templates nucleic acids extracted from three major bovine viruses (BCoV, BRSV, BPIV3) and two viruses known to cross-react with BVDV (BDV, CSFV). RNA from of BVDV-1, BVDV-2, and HoBiPeV were used as positive controls, and ddH_2_O served as the negative control.The results demonstrated that no amplification signals were observed for any of the five non-target viruses or the negative control.In contrast, specific amplification was observed exclusively for BVDV-1, BVDV-2, and HoBi-like pestivirus in their respective fluorescence channels, with no cross-reactivity detected for CSFV or BDV under the experimental conditions tested.These findings further confirm the high specificity and analytical reliability of the developed multiplex TaqMan RT-qPCR assay (Fig. 4).

### Sensitivity of the multiplex TaqMan RT-qPCR

**Fig. 5:**
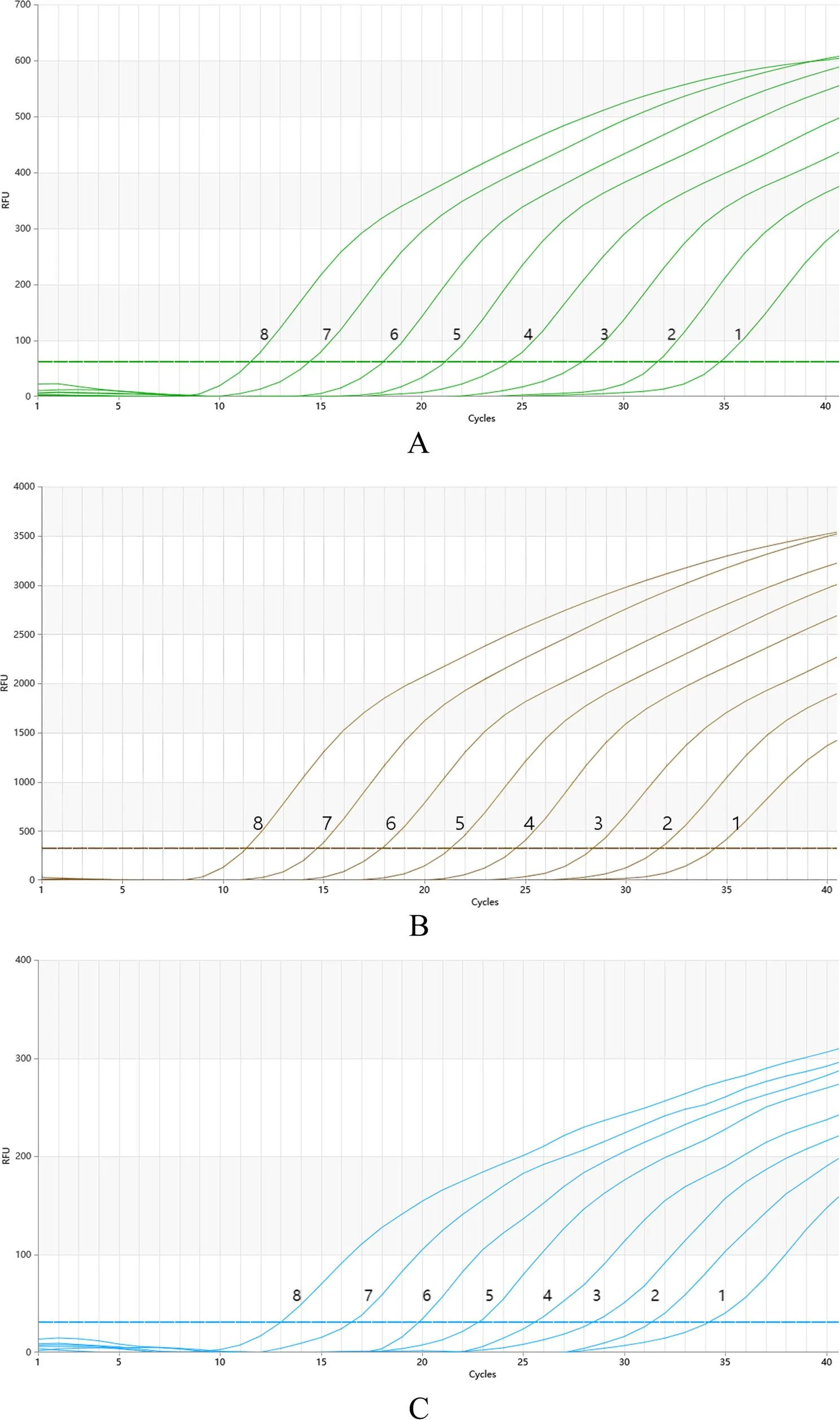
Assay sensitivity of the Multiplex RT-qPCR. **A** BVDV-1 5'UTR positive control; **B** BVDV-2 5'UTR positive control; **C** HoBiPeV 5'UTR positive control;The dilution series ranged from 10^1^ to 10^8^ (curves labeled 1-8 correspond to dilution factors of 10^1^, 10^2^, 10^3^, 10^4^, 10^5^, 10^6^, 10^7^, and 10^8^, respectively).Each amplification curve represents the real-time fluorescence signal plotted against the PCR cycle number, demonstrating the sensitivity and detection range of the multiplex RT-qPCR assay

To analyze the sensitivity of the multiplex TaqMan RT-qPCR method, we conducted tests with eight concentrations of standard reference RNA, which ranged from 1 × 10^1^ to 1 × 10^8^ copies/µL. The results indicate that the minimum detection limit and LOD for BVDV-1, BVDV-2, and HoBiPeV is the same at 1 × 10^1^ copies/µL (see Fig. 5A, B and C), which demonstrates high analytical sensitivity of the assay.According to the established result interpretation criteria, samples with Ct values < 35 and a typical S shaped amplification curve are considered positive. Samples with Ct ≥ 35, or with no Ct value and lacking a characteristic amplification curve, were interpreted as negative.

### Stability of the multiplex TaqMan RT-qPCR

To assess the repeatability of the multiplex TaqMan RT-qPCR assay, three replicate experiments were performed using mixed standard reference templates at concentrations of 1 × 10^4^, 1 × 10^5^, and 1 × 10^6^ copies/µL. The results showed that the intra-assay coefficient of variation (CV) ranged from 0.46% to 2.66%, while the inter-assay CV varied from 0.04% to 0.80%, thereby indicating robust reproducibility (Table 2).

**Table 2:** Repeatability analysis of multiplex RT-qPCR for Ct values

Plasmid	Concentration(Copies/µL)	Inter-Asssay			Intra-Asssay		
		Mean	S.D	CV	Mean	S.D	CV
BVDV-1	1 × 10^4^	30.46	0.20	0.67%	30.06	0.57	1.89%
	1 × 10^5^	27.29	0.01	0.04%	26.91	0.52	1.95%
	1 × 10^6^	23.01	0.02	0.07%	23.45	0.62	2.66%
BVDV-2	1 × 10^4^	30.28	0.04	0.13%	30.42	0.20	0.65%
	1 × 10^5^	27.75	0.22	0.80%	27.75	0.30	1.07%
	1 × 10^6^	24.01	0.07	0.28%	24.12	0.16	0.65%
HoBiPeV	1 × 10^4^	32.13	0.09	0.28%	32.37	0.34	1.04%
	1 × 10^5^	29.85	0.02	0.05%	29.51	0.48	1.63%
	1 × 10^6^	25.80	0.07	0.28%	25.88	0.12	0.46%

### Clinical sample testing

In this study, the established assay was used to test 176 clinical samples from Shaanxi Province. The results showed that 20 samples (20/176, 11.36%) were positive for BVDV-1, 10 samples (10/176, 5.68%) were positive for BVDV-2, and 2 samples (2/176, 6.81%) were positive for HoBiPeV. Using the method of Mari et al. (2016) as a comparative control, positive results were observed in 8 samples (8/176, 4.54%) for BVDV-1, 6 samples (6/176, 3.41%) for BVDV-2, and 1 samples (1/176, 0.57%) for HoBiPeV. Comparison of the two methods revealed discrepancies in 12 samples for BVDV-1, 4 samples for BVDV-2, and 1 samples for HoBiPeV.

Among the 20 BVDV-1 positive samples, all were detected by both the present method and the assay described by Mari et al., indicating high sensitivity. Five samples (F9, F57, F67, F74, and F81) were also positive by conventional RT-PCR and were confirmed as BVDV-1 by gene sequencing. In contrast, nine samples with high Ct values were detected only by the present method, while the Mari et al. assay, conventional RT-PCR, and sequencing were negative.Among the 10 BVDV-2 positive samples, four samples with relatively high viral loads (F73, F76, F83, and F85) were consistently detected by all three methods, and all except F83 were confirmed by sequencing. In samples with Ct values > 33, assay performance diverged: samples F16 and F65 were positive by both the present method and the Mari et al. assay but negative by conventional RT-PCR, whereas four samples with extremely low viral loads (F8, F24, F84, and F88) were detected exclusively by the present method.For the two HoBiPeV positive samples, F48 (Ct = 30.31) showed complete agreement among all methods, while F72 (Ct = 33.44) was detected by the present method and conventional RT-PCR but not by the Mari et al. assay, and sequencing was unsuccessful due to low viral concentration.(see Table 3 for details).

**Table 3:** Comparison of multiplex RT-qPCR, RT-PCR, and 5’ UTR sequencing results in clinical samples

Sample number	This study	Mari et al.	RT-PCR	Gene sequencing	Sample type
F8	2+(Ct:34.41)	-	-	/	Feces
F9	1+(Ct:25.4)	1+(Ct:26.2)	1/2+	1+	Feces
F15	1+(Ct:34.37)	1+(Ct:34.44)	-	/	Feces
F16	2+(Ct:33.49)	2+(Ct:33.96)	-	/	Feces
F17	1+(Ct:34.82)	-	-	/	Feces
F18	1+(Ct:33.43)	-	-	1+	Feces
F23	1+(Ct:34.74)	-	-	/	Feces
F24	2+(Ct:34.44)	-	-	/	Feces
F41	1+(Ct:34.02)	-	-	/	Feces
F48	HoBiPeV+(Ct:30.31)	HoBiPeV+(Ct:30.28)	HoBiPeV+	HoBiPeV+	Feces
F49	1+(Ct:34.06)	-	-	/	Feces
F50	1+(Ct:34.01)	-	-	/	Feces
F53	1+(Ct:34.21)	-	-	/	Feces
F54	1+(Ct:33.63)	-	-	1+	Feces
F57	1+(Ct:24.89)	1+(Ct:25.66)	1/2+	1+	Feces
F64	1+(Ct:32.71)	-	-	1+	Feces
F65	2+(Ct:33.96)	2+(Ct:34.7)	-	/	Feces
F67	1+(Ct:28.76)	1+(Ct:28.88)	1/2+	1+	Feces
F72	HoBiPeV+(Ct:33.44)	-	/	/	Feces
F73	2+(Ct:27.77)	2+(Ct:29.87)	1/2+	2+	Feces
F74	1+(Ct:31.2)	1+(Ct:32.03)	1/2+	1+	Feces
F76	2+(Ct:25.45)	2+(Ct:25.9)	1/2+	2+	Feces
F78	1+(Ct:34.83)	-	-	/	Feces
F79	1+(Ct:34.49)	-	-	/	nasal swabs
F80	1+(Ct:32.75)	1+(Ct:33.9)	-	/	nasal swabs
F81	1+(Ct:30.47)	1+(Ct:31.13)	1/2+	1+	nasal swabs
F83	2+(Ct:30.31)	2+(Ct:31.19)	1/2+	/	nasal swabs
F84	2+(Ct:34.16)	-	-	/	nasal swabs
F85	2+(Ct:30.22)	2+(Ct:30.73)	1/2+	2+	nasal swabs
F86	1+(Ct:34.42)	-	-	/	nasal swabs
F87	1+(Ct:33.8)	1+(Ct:33.96)	1/2+	/	nasal swabs
F88	2+(Ct:34.3)	-	-	/	nasal swabs

In summary, high concordance was observed among all methods for strongly positive samples, whereas the present method showed a clear advantage in weakly positive samples. The assay also performed well in both fecal and nasal swab specimens, indicating broad sample compatibility. Overall, parallel testing demonstrated that the multiplex TaqMan RT-qPCR outperformed conventional RT-PCR.Sequencing results were generally consistent with the detection assays, particularly in samples with low Ct values and high viral loads. Discrepancies between methods were mainly attributable to low viral loads (high Ct values), which often resulted in sequencing failure and reduced consistency.

## Discussion

BVDV is a major pathogenic agent that poses a serious threat to the global cattle industry, significantly impacting animal health and causing substantial economic losses.The three genotypes of BVDV-1, BVDV-2, and HoBiPeV exhibit significant differences in antigenicity and pathogenicity, which present challenges for clinical diagnosis and disease management [26].Extensive studies on BVDV-1 have supported the development of effective vaccines [27], whereas BVDV-2 infections are often associated with more severe outcomes, including thrombocytopenia, hemorrhaging, and increased mortality [28]. In recent years, HoBiPeV has attracted increasing attention due to its genomic divergence from BVDV-1 and BVDV-2 and its impact on viral transmission, epidemiology, and the effectiveness of existing vaccines and diagnostic methods [29, 30].The occurrence of HoBiPeV have mainly reported in South America, Europe, and Asia, indicating that its spread may be associated with international trade and animal transportation [31, 32]. However, methods for the simultaneous differentiation of these three genotypes remain limited, and identifying a suitable detection target site remains challenging [33–35].Therefore, developing a detection method that can simultaneously identify these three genotypes is crucial for the timely detection and control of BVDV transmission.

This study designed primers based on the alignment of the 5’ UTR regions of BVDV-1, BVDV-2, and HoBiPeV reference sequences published in the NCBI database. By incorporating a single degenerate nucleotide (Y = C/T) into the forward primer, the primer set achieves universal recognition capability for all three BVDV genotypes, enabling their concurrent detection and genotypic differentiation. The primer and probe set exhibits a high specificity for BVDV, with no observed cross-reactivity with BCoV, BRSV, BPIV 3, BDV or CSFV. In addition, the multiplex TaqMan RT-qPCR detection method established in this study exhibits exceptional performance across several key performance indicators. The method demonstrates a consistent minimum detection limit of 1 × 10¹copies/µL for the three representative in vitro-transcribed RNA standards tested, highlighting its efficacy in analyzing samples with low viral loads. Given that this detection limit was established using synthetic RNA standards, further validation with diverse viral isolates and clinical samples will be necessary to confirm its broader applicability.This is particularly crucial for early diagnosis and disease surveillance, as viral loads may be low during initial infection stages where conventional detection methods often fail to provide reliable identification. After the system was optimized, the R²values of the standard curves for the three representative BVDV species evaluated in this study were 1.00, 0.999, and 0.998, indicating high fitting accuracy and strong linearity across the tested concentration range. This enhancement is essential for epidemiological studies, facilitating precise assessments of viral transmission dynamics and infection severity in field settings.Additionally, the method demonstrated high amplification efficiencies for the three genotypes, indicating efficient target amplification and supporting reliable quantitative performance.While further validation using diverse field strains and clinical samples is warranted, such efficiency levels suggest that the assay has potential utility for quantitative applications.The method demonstrated intra-assay CVs ranging from 0.46% to 2.66%, as well as inter-assay CVs ranging from 0.04% to 0.80%, indicating exceptional repeatability and consistency in detection outcomes.

This stability is essential for large-scale detection efforts and epidemiological studies. The method utilizes a fully closed-tube operation, which not only prevents product contamination caused by the cap opening in traditional PCR but also prevents environmental pollution and reduces the occurrence of false-positive results. The entire detection process is simple and only requires 1 to 2 hours, significantly enhancing clinical testing efficiency and enabling timely provision of critical data to support clinical decision-making. Compared to conventional PCR methods, the results are visually straightforward and presented in a more objective data format, facilitating clear differentiation of samples as positive, negative, or equivocal, thus ensuring the accuracy of the findings. The comparative analysis of clinical samples revealed that the positive rates were 11.36% (20/176) for BVDV-1, 5.68% (10/176) for BVDV-2, and 0.57% (1/176) for HoBiPeV. The present assay demonstrated high detection rates for all three target genotypes, with particularly strong analytical sensitivity for BVDV‑1.This difference may be attributed to the degenerate primers (Y = C/T) designed in this method, which covered the variation sites in the 5’ UTR region of the BVDV-2 and HoBiPeV genomes. High concordance was observed among all methods for strongly positive samples, whereas the present method showed a clear advantage in weakly positive samples. In addition, the assay performed well in both fecal and nasal swab specimens, indicating broad sample compatibility. Overall, parallel testing demonstrated that the multiplex TaqMan RT‑qPCR outperformed conventional RT‑PCR.Sequencing results were generally consistent with RT‑qPCR typing, particularly in samples with low Ct values and high viral loads. However, sequencing failures were frequently observed in samples with high Ct values, indicating that conventional RT‑PCR–based sequencing has limited sensitivity when viral loads are low.

Despite the substantial advantages demonstrated by this method regarding sensitivity and specificity, two critical issues must still be considered in its application. Firstly, the increased detection sensitivity implies that trace nucleic acid contamination may lead to false-positive results, with nucleic acid aerosols being a common source of contaminants in laboratories. The sensitivity of the detection system to nucleic acid aerosol contamination requires laboratories to establish strict zoning management protocols and maintain continuous environmental monitoring programs. Secondly, given the continuous mutation of virus genome, it is necessary to assess the binding efficiency of the primer-probe in a timely manner to enhance primer adaptability.At the same time, when whole-genome sequencing is not feasible, the 5’ UTR targeted in this study can serve as a conserved region for rapid preliminary screening and genotype assignment [23].However, the 5’ UTR has limited length and sequence diversity; because it contains few informative sites, it cannot reliably resolve relationships among major clades.Therefore, we recommend using the full N^pro^ and E2 coding regions as supplemental or alternative targets for phylogenetic inference and genotyping.The N^pro^ and E2 regions contain much more variable information and are more advantageous for sub-genotypes delineation and lineage resolution. Practical strategies include: using the 5’ UTR as the primary target for routine rapid typing; for RT-qPCR positive samples that require further sub-genotypes or trace-back, adding amplification and sequencing of N^pro^ or E2; and prioritizing multi-region combined sequencing in study or surveillance designs (for example 5’ UTR+N^pro^+E2) to balance detection sensitivity and phylogenetic resolution within available resources.The method established in this study successfully achieved precise identification and detection of three genotypic strains: BVDV-1, BVDV-2, and HoBiPeV. The results of the clinical testing indicate that this method not only significantly enhances the detection rate of HoBiPeV but also elucidates the potential prevalence of this genotype within cattle populations. These findings are of considerable practical significance for the updating of epidemiological databases and the selection of vaccine strains.

## Conclusion

This study successfully established and validated a multiplex TaqMan RT-qPCR detection method that can simultaneously detect and differentiate BVDV-1, BVDV-2, and HoBiPeV, particularly the newly identified HoBiPeV in recent years.The assay demonstrated high specificity, sensitivity, and reliable performance under the experimental conditions applied. Although further validation with diverse field strains and larger clinical sample sets is needed, this method provides a useful platform for future diagnostic and epidemiological studies of BVDV infections.

## Methods

### Virus isolates and clinical samples

The BVDV-1 strain NADL, BVDV-2 strain New York, and HoBiPeV strain Th/04_KhonKaen, as well as positive nucleic acid samples of Bovine Coronavirus(BCoV)、Bovine Respiratory Syncytial virus (BRSV) Bovine Parainfluenza Virus Type 3 (BPIV3) Border disease virus (BDV) and Classic Swine Fever Virus (CSFV) were provided by the China Animal Health and Epidemiology Center (Qingdao, China). In this study, these viral samples and positive nucleic acids were used for the construction of recombinant plasmids as templates for subsequent in vitro transcription of RNA standards, as well as for analytical specificity evaluation. A total of 176 clinical samples, including fecal samples and nasal swabs, were collected from cattle farms in Shanxi Province, China.

### Primers and probes design

The 5’UTR regions of reference strains of BVDV-1, BVDV-2, and HoBiPeV were retrieved from the NCBI database (Table 4). Sequence alignments were performed using MegAlign (DNASTAR, USA) with the ClustalW algorithm, using default parameters, with emphasis on recently circulating HoBiPeV. reference strains.In parallel, sequence homology analysis of the reference sequences was performed with the SDTv1.3 software to identify conserved regions of BVDV-1, BVDV-2, and HoBiPeV.Based on the conserved regions of the 5’UTR of BVDV-1, BVDV-2, and HoBiPeV, we designed a pair of universal primers (applicable to all three genotypes) using Primer Premier 5 (Premier Biosoft International, Palo Alto, CA, USA), along with three genotype-specific TaqMan probes. The specificity of primers and probes was verified using the NCBI BLAST tool. The sequences of the primers and probes are listed in Table 4 and were synthesized by Sangon Biotech (Shanghai).

**Table 4:** List of reference strains for primer and probe design

Genotype	Subtype	Strain	Origin	Location	Collection year	5’UTR Accession number
BVDV-1	1a	NADL	Cattle	USA	1963	AJ133739
BVDV-1	1a	Oregon C24 V	Cattle	USA	1998	AF091605.1
BVDV-1	1a	BJ-2013	Cattle	China	2013	MH490942.1
BVDV-1	1a	SWU-Z6	Fecal	China	2016	MF693403
BVDV-1	1a	MRI1354	Bos taurus	UK	2017	LT901719.1
BVDV-1	1a	62 − 2	Cattle	UK	2008	MW250798.1
BVDV-1	1b	BJ-2016	Cattle	China	2016	MH490943.1
BVDV-1	1b	12F004	Cattle	South Korea	2012	KC963967.1
BVDV-1	1b	3156	Cattle	China	2011	JN704144
BVDV-1	1c	BJ175170	Cattle	China	2017	MT119454.1
BVDV-1	1c	MF-1	Cattle	China	2016	KY865369
BVDV-1	1c	Bega-like	Bovine	Australia	2012	KF896608
BVDV-1	1c	NM2103	Cattle	China	2021	ON337882
BVDV-1	1c	NSWp5	Bos taurus	Australia	2019	OP020142.1
BVDV-1	1d	10-JJ-SKR	Cattle	South Korea	2010	KC757383
BVDV-1	1d	SWU-DJ2	Yak	China	2015	MF166858
BVDV-1	1e	68 − 1	Cattle	UK	2008	MW250802
BVDV-1	1e	S03-1175	Cattle	USA	2003	MW655631
BVDV-1	1i	ACM/BR/2016	Cattle	Brazil	2017	KX857724
BVDV-1	1 m	SD-15	Cattle	China	2015	KR866116
BVDV-1	1 m	CHN/HB-01/2017	Bovine	China	2017	ON901784
BVDV-1	1 m	NM2312	Cattle	China	2023	PP992316.2
BVDV-1	1o	HA2-12	Goat	China	2013	KP749802
BVDV-1	1o	MF-3	Cattle	China	2016	KY865371
BVDV-1	1o	XH-2	Cattle	China	2016	KY865375
BVDV-1	1o	IS26NCP/01	Cattle	Japan	2007	AB359932
BVDV-2	-	890	-	USA	1997	U18059.1
BVDV-2	-	NewYork’93	-	USA	2002	AF502399.1
BVDV-2	2a	HLJ-10	Cattle	China	2011	JF714967
BVDV-2	2a	296c	-	USA	1995	MH806436.1
BVDV-2	2b	SD1301	Cattle	China	2012	KJ000672
BVDV-2	2c	SH2210-23	Cattle	Germany	2010	HG426494
BVDV-2	-	Hokudai-Lab09	-	Japan	2010	AB567658.1
BVDV-2	-	XJ-04	Cattle	China	2004	NC-076032.1
HoBiPeV	3	XJ-BVDV-3	Bovine	China	2022	OP210314.1
HoBiPeV	3	D3200-HoBi	Bos taurus	Brazil	2004	AB871953.1
HoBiPeV	3	Italy-1/10 − 1	Cattle	Italy	2010	HQ231763.1
HoBiPeV	3	JS12/01	Bovine	China	2012	JX469119.1
HoBiPeV	3	LVRI/cont-1	Bovine	South America	2012	KC297709.1
HoBiPeV	3	HN1507	Capra hircus	China	2015	KU563155.1
HoBiPeV	3	SV757-15	Bos taurus	Brazil	2015	KY683847.1
CSFV	CSFV	Shimen	Swine	China	1999	AF092448.2
CSFV	CSFV	SXCDK	Swine	China	2009	GQ923951
BDV	BDV	JSLS12-01	Ovis aries	China	2012	KC963426.1
BDV	BDV	X818	Sheep	Germany	1997	NC_003679.1

### Construction of standard plasmids

Total RNA was extracted from the representative reference strains BVDV-1 NADL, BVDV-2 New York, and HoBiPeV Th/04_KhonKaen, and reverse transcribed into cDNA. The 5’ UTR region of reference sequences was subsequently amplified by RT-PCR using primers BVDV-1/2-TF, BVDV-HoBiPeV-F, and BVDV-1/2/HoBiPeV-TR (Table 1). The amplified 5’ UTR fragments were cloned into the pUC-57 vector (TaKaRa, Qingdao, China), resulting in the construction of recombinant plasmids, including pUC-57-BVDV-1-5’UTR, pUC-57-BVDV-2-5’UTR, and pUC-57-HoBiPeV-5’UTR, which were used exclusively as DNA templates for subsequent in vitro transcription. Subsequently, the plasmids were transformed into E. coli DH5α (TaKaRa, Dalian, China).Positive clones were cultured for amplification and plasmid extraction. After linearization by single-enzyme digestion, the linearized plasmids were recovered and purified from agarosegel, and their concentrations were measured with a NanoDrop spectrophotometer (Thermo Fisher, Waltham, MA, USA). The linearized plasmids containing a T7 promoter were then used as templates for in vitro transcription to generate RNA standards corresponding to the 5’UTR of BVDV-1, BVDV-2, and HoBiPeV, using T7 RNA polymerase (Accurate Biology, Changsha, China).The transcribed RNA was purified, quantified by NanoDrop spectrophotometry, and the RNA copy number was calculated based on transcript length and concentration. These RNA transcripts were used as standards for the establishment of standard curves and for determining the sensitivity of the RT-qPCR assay.Purified RNA standards were aliquoted and stored at − 80 ℃ until use.

### Optimization of multiplex TaqMan RT-qPCR detection method

The three in vitro-transcribed RNA standards, were diluted to concentrations ranging from 1 × 10^1^ to 1 × 10^8^ copies/µL and mixed in equal proportions to be used as amplification templates for multiplex TaqMan RT-qPCR optimization. Using the mixed RNA standards as templates, primers and three probe pairs were combined in a single reaction system for simultaneous detection of RNA targets of BVDV-1, BVDV-2, and HoBiPeV. To explore the optimal conditions for this multiplex reaction, a systematic optimization of primer and probe concentrations as well as annealing temperature was conducted. The optimized multiplex TaqMan RT-qPCR amplification system (total volume of 20 µL) is as follows: 4 µL of U + One Step RT-qPCR Probe 5×Master Mix(Vazyme, Nanjing, China), 0.2 µL of BVDV-1/2/HoBiPeV-F (10 µM), 0.2 µL of BVDV-1/2/HoBiPeV-R (10 µM), 0.2 µL of BVDV-1-P (10 µM), 0.4 µL of BVDV-2-P (10 µM), 0.2 µL of BVDV-HoBiPeV-P (10 µM), 11.8 µL of RNase-free ddH_2_O, and 3 µL of RNA standard template.The amplification procedure included 55℃ for 15 min, 95℃ for 30 s, followed by 35 ~ 40 cycles of 95℃ for 10 s and 60℃ for 30 s.During the annealing phase, fluorescent signals were collected through the HEX, ROX, and FAM channels to generate amplification curves for RNA-based standard curve construction.

### Establishment of a multiplex TaqMan RT-qPCR standard curve

A 10-fold serial dilution of mixed in vitro-transcribed RNA standards derived from representative reference sequences of BVDV-1, BVDV-2, and HoBiPeV 5’UTR regions was prepared with concentrations spanning from 1 × 10^1^ to 1 × 10^8^ copies/µL and used as templates for amplification.These RNA standards were generated from plasmids containing the target regions and were used as quantification standards for assay development and evaluation of amplification efficiency.For each dilution point, perform three technical replicates using the established multiplex TaqMan RT-qPCR assay.The log_10_ of the RNA copy number was plotted on the horizontal axis (x-axis), and the corresponding Ct values obtained from RT-qPCR amplification of RNA templates were plotted on the vertical axis (y-axis).RNA-based quantitative standard curves were generated for BVDV-1, BVDV-2, and HoBiPeV. These standard curves were intended for analytical performance evaluation during assay development.

### Analysis of specificity, sensitivity, and repeatability in multiplex TaqMan RT-qPCR

To evaluate the specificity of the assay, multiplex TaqMan RT-qPCR was performed under optimized reaction conditions using nontarget RNA extracted from nontarget bovine viruses, including BCoV, BRSV, BPIV3 as templates.CSFV and BDV samples were included in specificity testing, with reference to WOAH-recommended diagnostic targets for pestivirus differentiation.These viruses are all RNA viruses, and the RNA input amounts were within the concentration range commonly used for routine diagnostic RT-qPCR assays, corresponding to midrange amplification levels (approximately Ct 20–30).A mixed in vitro-transcribed RNA standard (1 × 10^5^ copies/µL) containing BVDV-1, BVDV-2, and HoBiPeV targets was used as a positive control, while nuclease-free water served as a negative control.To evaluate the sensitivity of the kit, a mixed in vitro-transcribed RNA standard was used as the template, and ten-fold serial dilutions ranging from 1 × 10^8^ to 1 × 10^1^ copies/µL were prepared for RT-qPCR testing. The limit of detection (LOD) was determined from replicate results across the dilution series as the lowest concentration reliably detected at the specified confidence level.To evaluate the stability of the kit, four concentrations of the mixed in vitro-transcribed RNA standard (1 × 10^8^, 1 × 10^7^, 1 × 10^6^, and 1 × 10^5^ copies/µL) were used, with three intra-assay and inter-assay replicates performed for each concentration.

### Multiplex TaqMan RT-qPCR detection of clinical samples

A total of 176 clinical samples (including feces and nasal swabs) were collected from Shaanxi regions in China. Viral total nucleic acids were extracted using the Rapid Nucleic Acid Extraction Kit (Xi’an TIANLONG Technology Co, Ltd, Shaanxi, China). In vitro‑transcribed RNA standards were used as positive controls and ddH_2_O as negative controls, followed by detection via the multiplex TaqMan RT-qPCR method. Concurrently, the same samples were tested in parallel using the method described by Mari et al. (2016) to compare the detection results of the two methods.In addition, all samples were tested by RT-PCR targeting the 5’UTR using primer BVDV-1/2-TF, BVDV-1/2/HoBiPeV-TR, and HoBiPeV-TF (Table 1). All PCR-positive products were sequenced, and the sequences were submitted to NCBI GenBank for comparative analysis to confirm the genotypes.

## Data Availability

All the data supporting the results in the current study is contained within the manuscript.
